# Large−scale SSR fingerprinting resolves taxonomic distinction between *Picea purpurea* and *Picea asperata* and reveals elevation−linked diversity in Qinghai Province, China

**DOI:** 10.3389/fpls.2026.1882848

**Published:** 2026-07-10

**Authors:** Yinyan Qi, Bo Mu, Xu Lu, Dongcuoji Li, Longhua Xing, Wenyi Gu

**Affiliations:** 1 Qinghai Academy of Agriculture and Forestry Sciences, Qinghai University, Xining, Qinghai, China; 2 Qinghai Plateau Key Laboratory of Tree Genetics and Breeding, Xining, Qinghai, China; 3 Research and Utilization Laboratory of Qinghai-Tibet Plateau Germplasm Resources, Xining, Qinghai, China; 4 Xiaqun Forest Farm of Ping’an, Haidong, Qinghai, China

**Keywords:** germplasm conservation, *Picea*, population structure, SSR fingerprinting, taxonomic resolution

## Abstract

Spruce (*Picea* spp.) are ecologically and economically important conifers. However, species delimitation within the genus remains challenging due to extensive morphological plasticity and interspecific gene flow. In this study, we developed a high-resolution molecular identification system based on 15 fluorescent simple sequence repeat (SSR) markers and applied it to 516 spruce individuals representing 13 natural populations across Qinghai Province, China. The SSR panel demonstrated exceptional discriminatory capacity, with a discrimination power (DP) of 0.9998 and an extremely low probability of identity (PI) of 2.4 × 10^-5^, enabling accurate individual fingerprinting and germplasm traceability. Population structure analyses, including STRUCTURE, principal coordinate analysis (PCoA), and phylogenetic reconstruction, consistently resolved two highly divergent genetic lineages corresponding to *Picea purpurea* and *P. asperata*. These results provide strong molecular support for their taxonomic distinctiveness, as evidenced by substantial genetic differentiation (FST = 0.324–0.453) and limited gene flow (Nm < 0.11). In contrast, populations of *P. asperata* exhibited low levels of genetic differentiation (FST = 0.004–0.107) and extensive admixture, indicating considerable historical and contemporary gene flow among populations. Notably, we identified a significant negative correlation between expected heterozygosity (He) and elevation (r=−0.67, *p* = 0.012), revealing an elevation−associated genetic diversity cline in which high−elevation populations harbor significantly reduced genetic diversity, a pattern likely reflecting smaller effective population sizes and historical demographic bottlenecks at higher altitudes. This finding underscores the utility of SSR markers in detecting environmentally associated patterns of genetic variation: despite being selectively neutral, their high polymorphism and genome−wide distribution, combined with extensive sampling (516 individuals) across a broad elevational gradient (2,120–3,600 m), provided sufficient resolution to reveal this ecologically meaningful cline. Overall, this study establishes a robust SSR-based fingerprinting framework for spruce germplasm identification and management, resolves longstanding taxonomic uncertainty between *P. purpurea* and *P. asperata*, and provides novel insights into elevation-associated genetic variation. These findings have important implications for conservation prioritization, germplasm resource management, and the development of climate-resilient forest management strategies in the Qinghai–Tibet Plateau region.

## 1 Introduction

Spruce (*Picea* spp.) represents one of the most ecologically dominant and economically important conifer genera in boreal and montane forests throughout the Northern Hemisphere. As foundation species of many forest ecosystems, spruces provide a wide range of ecosystem services, including carbon sequestration, biodiversity conservation, soil stabilization, hydrological regulation, and climate buffering (Cordaro et al., 2025; Rahman et al., 2021). In addition to their ecological significance, spruce species are major sources of timber, pulpwood, and planting materials used in afforestation, ecological restoration, and sustainable forest management programs. Owing to their extensive geographic distribution and long evolutionary history, *Picea* species have become important model systems for investigating speciation, adaptation, demographic dynamics, and the genetic consequences of environmental change.

China is recognized as one of the major centers of diversity for the genus *Picea*, harboring more than 20 native species, many of which are endemic to the Qinghai–Tibet Plateau (QTP) and adjacent mountainous regions. The QTP has undergone dramatic geological uplift and recurrent climatic oscillations throughout the Quaternary, generating pronounced environmental heterogeneity and complex biogeographic patterns. These processes have facilitated population isolation, secondary contact, hybridization, and adaptive divergence among forest tree species, making the region an exceptional natural laboratory for studying evolutionary diversification and speciation in conifers. Previous phylogenetic and genomic investigations have demonstrated that the complex topography and climatic history of the QTP have profoundly influenced the evolutionary trajectories, geographic distributions, and genetic structures of spruce species (Lockwood et al., 2013; Ran et al., 2018a; Shen et al., 2019a).

Despite extensive taxonomic research, species delimitation within *Picea* remains challenging. Diagnostic morphological traits often overlap among closely related taxa and may be strongly affected by environmental conditions, resulting in substantial phenotypic plasticity. Moreover, natural hybridization and historical introgression are widespread within the genus, frequently obscuring species boundaries and generating conflicting taxonomic interpretations (Sullivan et al., 2017; Ru et al., 2018). Recent transcriptomic analyses have further revealed complex patterns of adaptive divergence between closely related spruce species on the QTP, underscoring the need for high-resolution molecular markers to resolve species boundaries (Feng et al., 2023). Such uncertainty not only limits our understanding of spruce evolutionary history but also constrains effective conservation planning, breeding programs, and sustainable utilization of genetic resources.

A representative example is *Picea purpurea*, an endemic spruce species distributed primarily in the eastern Qinghai–Tibet Plateau. Its taxonomic status and evolutionary origin have long been debated because of its morphological similarity to the more widely distributed *Picea asperata*. While some studies have recognized *P. purpurea* as an independent evolutionary lineage, others have suggested that it may have originated through historical hybridization or represent a derivative of the *P. asperata* complex. Resolving this taxonomic ambiguity is particularly important because inaccurate species identification may lead to biased assessments of genetic diversity, inappropriate germplasm management strategies, and ineffective conservation measures. Therefore, robust molecular evidence is required to clarify species boundaries and support evidence-based conservation of spruce genetic resources in the QTP region.

Molecular markers have become indispensable tools for species identification, germplasm characterization, and population genetic analyses. Among available marker systems, simple sequence repeats (SSRs) are particularly advantageous because of their codominant inheritance, high polymorphism, reproducibility, and broad applicability across diverse plant taxa (Morgante et al., 1996; Si et al., 2022). Consequently, SSR markers have been widely employed in studies of genetic diversity, population structure, parentage analysis, and molecular fingerprinting in forest trees, including several *Picea* species. Previous studies have demonstrated their utility in assessing genetic variation and population differentiation in spruce populations (Wang et al., 2006; Aizawa et al., 2009). Nevertheless, most investigations have focused primarily on population diversity and structure, whereas the development of practical DNA fingerprinting systems for large-scale germplasm management remains limited.

Despite growing interest in the evolutionary history and genetic diversity of Chinese spruces, several important knowledge gaps remain. In particular, no comprehensive SSR-based study has systematically compared *Picea purpurea* and *Picea asperata* across a broad geographic scale, and molecular evidence clarifying their taxonomic relationship is still insufficient. Furthermore, robust DNA fingerprinting platforms suitable for germplasm authentication, provenance verification, and genetic resource traceability are lacking for spruce populations in Qinghai Province. Such limitations hinder the efficient conservation and utilization of valuable germplasm resources, especially under increasing pressures from habitat fragmentation, ecological restoration initiatives, and ongoing climate change.

To address these gaps, we analyzed 516 *Picea* accessions representing 13 natural population groups across Qinghai Province using 15 highly polymorphic SSR loci. Specifically, our objectives were to: (1) develop and validate a high-resolution DNA fingerprinting system for accurate germplasm identification and genetic traceability; (2) clarify the taxonomic relationship between *Picea purpurea* and *Picea asperata* using population genetic and phylogenetic approaches; (3) characterize genetic diversity, population structure, and admixture patterns among *P. asperata* populations; and (4) evaluate potential ecogenetic associations. We hypothesized that *P. purpurea* represents a genetically distinct evolutionary lineage separated from *P. asperata*, whereas *P. asperata* populations exhibit relatively weak genetic differentiation due to extensive gene flow. By integrating molecular fingerprinting and population genetic analyses, this study provides new insights into spruce evolution, species delimitation, and population connectivity while establishing practical tools for the conservation and sustainable management of spruce genetic resources in the Qinghai–Tibet Plateau.

## 2 Materials and methods

### 2.1 Plant materials and sampling design

A total of 516 spruce accessions representing 13 natural population groups were sampled across the major distribution ranges of spruce in Qinghai Province, China (**Supplementary Table 1**; **Supplementary Figure 1**). To capture the broad geographic, ecological, and environmental heterogeneity of the region, a stratified random sampling strategy was implemented following principles commonly applied in population genetics and landscape genomics studies (Holderegger and Wagner, 2006; Storfer et al., 2007). First, geographic stratification was conducted using a 0.5 × 0.5 grid system covering all major spruce distribution areas within Qinghai Province. Sampling sites were selected to ensure comprehensive representation of the species’ spatial distribution while minimizing geographic sampling bias. This approach facilitated the evaluation of spatial genetic patterns and provided sufficient resolution for subsequent analyses of isolation-by-distance (IBD) and population connectivity (Wright, 1943; van Strien et al., 2015).

Second, ecological stratification was performed based on elevation and slope aspect, two environmental factors known to strongly influence forest ecosystem dynamics and local adaptation in alpine tree species. Three elevation classes were defined: low elevation (<2800 m), mid elevation (2800–3200 m), and high elevation (>3200 m). Within each elevation zone, sampling was further balanced between north-facing and south-facing slopes whenever natural populations were available. This design enabled the incorporation of environmental gradients into downstream genotype–environment association and ecological differentiation analyses (Storfer et al., 2007; Bishop et al., 2025).

To ensure that sampled populations represented naturally established genetic resources, only natural stands containing more than 50 mature individuals were included in the study. Artificial plantations, recently established stands, and heavily disturbed populations were excluded to minimize the influence of anthropogenic factors on genetic structure. Within each population, trees were selected randomly while maintaining a minimum distance of 50 m between sampled individuals. This criterion reduced the likelihood of repeatedly sampling closely related individuals, root-connected trees, or potential clonal ramets, thereby improving the accuracy of population genetic parameter estimation. For each sampled tree, geographic coordinates, elevation, slope aspect, and habitat information were recorded using a handheld Global Positioning System (GPS) device. Young, healthy needles were collected, immediately preserved, and transported to the laboratory for DNA extraction.

### 2.2 DNA extraction, SSR marker development, and genotyping

Fresh needle tissues were collected from each accession and stored at −80 °C. Genomic DNA was isolated using a standard CTAB protocol (Doyle and Doyle, 1987). DNA quality and concentration were evaluated using 1% agarose gel electrophoresis and a NanoDrop 2000c spectrophotometer (Thermo Fisher Scientific, USA). Chemicals: Agarose (Biowest, Spain), Tris base and boric acid (Sigma-Aldrich, USA), GelRed (Biotium, USA), DNA ladder and loading buffer (Takara Bio, Japan). Instruments: NanoDrop 2000c (Thermo Fisher Scientific, USA), Sub-Cell GT electrophoresis system and GelDoc XR+ documentation system (Bio-Rad, USA).

SSR marker development: SSR loci were identified from our previously generated full-length transcriptome data of spruce (available at https://doi.org/10.6084/m9.figshare.32677611) using MISA (MicroSatellite identification) software (Thiel et al., 2003). The parameters for microsatellite definition were set as follows: unit size and minimum number of repeats: (1/20) (2/6) (3/5) (4/5) (5/5) (6/5); maximal number of bases interrupting two SSRs in a compound microsatellite = 100. This analysis identified a total of 450,157 SSR loci across the transcriptome assembly.

From this large SSR pool, 192 candidate loci were selected for primer design based on criteria including: (i) repeat motif type (di−, tri−, and tetranucleotide repeats preferred), (ii) repeat number (≥6 for di−nucleotide, ≥5 for tri− and tetra−nucleotide), (iii) suitability of flanking sequences for primer design, and (iv) predicted amplicon size (100–300 bp). Primer pairs were designed using Primer3 (Untergasser et al., 2012) with default parameters.

Polymorphism screening: To evaluate polymorphism and amplification reliability, all 192 candidate primer pairs were initially tested on a panel of 13 DNA samples representing genetically divergent individuals from different populations and species. PCR products were resolved on 2% agarose gels to confirm amplification success, and polymorphic markers were further evaluated using capillary electrophoresis on an ABI 3730xl DNA Analyzer (Applied Biosystems, USA). Markers were retained if they met the following criteria: (i) clear and reproducible amplification in >90% of test samples, (ii) presence of polymorphism (≥2 alleles) across the test panel, (iii) absence of stutter peaks or nonspecific amplification, and (iv) consistent peak patterns across replicate runs.

Final marker set: This screening process yielded 15 highly polymorphic and reproducible SSR loci, which were subsequently used for genotyping all 516 accessions. The final primer details (sequences, repeat motifs, annealing temperatures, fluorescent labels, and sources) are provided in **Supplementary Table 2**.

SSR genotyping: Fluorescent PCR amplification was performed using the M13-tailed primer labeling method (Schuelke, 2000) in a 20 μL reaction containing 30 ng genomic DNA, 0.2 μM each of forward (with M13 tail), reverse, and fluorescently labeled M13 primers, 0.2 mM dNTPs, 1.5 mM MgCl_2_, and 1 U Taq polymerase (Takara Bio, Japan). Thermal cycling conditions were: 94 °C for 3 min; 35 cycles of 94 °C for 30 s, annealing at optimized temperatures (54–58 °C, see **Supplementary Table 2**) for 30 s, 72 °C for 45 s; and a final extension at 72 °C for 10 min. Amplified fragments were separated by capillary electrophoresis on an ABI 3730xl DNA Analyzer (Applied Biosystems, USA). Fragment sizes were determined using GeneMapper v5.0 (Applied Biosystems) against an internal size standard (LIZ500).

### 2.3 DNA fingerprinting and validation

DNA fingerprints were generated for all 516 spruce accessions based on the allelic profiles obtained from the 15 selected SSR loci. For each accession, a binary matrix was constructed by scoring the presence (1) or absence (0) of each detected allele across all loci. The resulting multilocus allelic profile was defined as the DNA fingerprint of the corresponding accession (**Supplementary Table 3**).

To evaluate the effectiveness and reliability of the fingerprinting system, several statistical parameters commonly used in individual identification studies were calculated using GenAlEx version 6.5 (Peakall and Smouse, 2012). These parameters included discrimination power (DP), which estimates the probability that two randomly selected individuals possess different multilocus genotypes, and the probability of identity (PI), which represents the probability that two unrelated individuals share an identical multilocus genotype by chance. In addition, the probability of identity among siblings (PI_sibs) was calculated as a more conservative measure of individual discrimination, particularly for populations with potential relatedness among sampled individuals (Waits et al., 2001).

To assess the independence of SSR loci and verify their suitability for multilocus fingerprint construction, pairwise linkage disequilibrium (LD) tests were performed among all locus pairs using GENEPOP version 4.7 (Rousset, 2008). Statistical significance was evaluated after Bonferroni correction for multiple comparisons. Only loci exhibiting no significant linkage disequilibrium were considered independent and suitable for inclusion in the final DNA fingerprinting system.

### 2.4 Population structure and phylogenetic analysis

Population genetic structure was inferred using the Bayesian clustering approach implemented in STRUCTURE version 2.3.4 (Pritchard et al., 2000). Analyses were performed under an admixture model with correlated allele frequencies. The number of genetic clusters (K) was evaluated from K = 1 to K = 20, with 20 independent runs conducted for each K value. Each run consisted of a burn-in period of 10,000 iterations followed by 100,000 Markov chain Monte Carlo (MCMC) iterations. The most likely number of genetic clusters was determined using the ΔK method (Evanno et al., 2005), as implemented in STRUCTURE HARVESTER version 0.6.94 (Earl and vonHoldt, 2012).

To further investigate genetic relationships among accessions, a neighbor-joining (NJ) phylogenetic tree was constructed based on Nei’s unbiased genetic distance (Nei, 1978). Tree construction and visualization were performed using MEGA X version 10.2.6 (Kumar et al., 2018). The NJ method was selected because it provides an efficient and robust approach for reconstructing genetic relationships from multilocus SSR data and has been widely applied in population genetic studies of conifer species. The reliability of tree topology was evaluated using 1,000 bootstrap replicates.

### 2.5 Principal coordinate analysis

Principal coordinate analysis (PCoA) was conducted using GenAlEx version 6.5 (Peakall and Smouse, 2012) based on a codominant genetic distance matrix generated from all 15 SSR loci. The first two principal coordinates, which explained the largest proportion of the genetic variation, were extracted and visualized using R version 4.2.1 (R Core Team, 2022; Excoffier and Lischer, 2010) with the ggplot2 package (Wickham, 2016; Jombart, 2008).

### 2.6 Genetic diversity, differentiation, and ecogenetic analyses

Genetic diversity parameters, including the number of alleles (Na), effective number of alleles (Ne), observed heterozygosity (Ho), expected heterozygosity (He), Shannon’s information index (I), and fixation index (F), were calculated for each population and locus using GenAlEx v6.5 (Peakall and Smouse, 2012). Polymorphism information content (PIC) was estimated using CERVUS v3.0 (Kalinowski et al., 2007).

Genetic differentiation among populations was assessed by calculating pairwise fixation indices (FST) in FSTAT v2.9.4 (Goudet, 2003). Gene flow (Nm) was estimated from FST values according to the equation Nm = (1/FST − 1)/4. Analysis of molecular variance (AMOVA) was conducted in GenAlEx v6.5 with 9,999 permutations to partition genetic variation among species, among populations within species, and within populations.

Isolation by distance (IBD) was evaluated using Mantel tests implemented in GenAlEx v6.5 with 9,999 permutations. Pairwise genetic differentiation [FST/(1 − FST)] was correlated with the natural logarithm of geographic distance (km) between population pairs. To assess environmental effects on genetic differentiation, partial Mantel tests were performed by correlating pairwise genetic distances with pairwise environmental distances derived from elevation.

## 3 Results

### 3.1 Genetic diversity and SSR polymorphism

A total of 15 simple sequence repeat (SSR) loci were used to assess genetic diversity across 516 accessions. All loci exhibited a high level of polymorphism, demonstrating their suitability for population genetic analyses. The number of alleles per locus (Na) ranged from 2.000 to 10.000, indicating substantial allelic richness across the studied germplasm (**Table 1**).

**Table 1:** Polymorphism information of 15 pairs of SSR loci.

locus	Na	Ne	I	Ho	He	F	PIC	Prob	Signif
YSSSR032	6.000	1.665	0.734	0.292	0.399	0.268	0.356	0.000	***
YSSSR063	8.000	1.421	0.614	0.217	0.296	0.268	0.276	0.000	***
YSSSR074	6.000	1.505	0.653	0.307	0.336	0.086	0.302	0.000	***
YSSSR077	5.000	1.873	0.883	0.282	0.466	0.394	0.420	0.000	***
YSSSR092	5.000	2.512	1.040	0.495	0.602	0.177	0.520	0.000	***
YSSSR096	10.000	1.839	1.072	0.327	0.456	0.282	0.440	0.000	***
YSSSR099	2.000	1.967	0.685	0.420	0.492	0.146	0.371	0.001	***
YSSSR111	4.000	1.781	0.788	0.316	0.438	0.279	0.389	0.000	***
YSSSR118	8.000	1.366	0.633	0.215	0.268	0.198	0.259	0.000	***
YSSSR130	3.000	1.476	0.606	0.218	0.323	0.325	0.297	0.000	***
YSSSR142	8.000	2.250	0.995	0.491	0.556	0.116	0.472	0.262	ns
YSSSR171	2.000	1.945	0.679	0.335	0.486	0.311	0.368	0.000	***
YSSSR002	4.000	1.914	0.780	0.636	0.478	-0.331	0.397	0.000	***
YSSSR051	5.000	1.798	0.828	0.468	0.444	-0.055	0.397	0.533	ns
YSSSR072	6.000	1.245	0.430	0.086	0.197	0.562	0.188	0.000	***
Mean	5.467	1.770	0.761	0.340	0.416	0.202	0.363	0.053	
STDEV	2.326	0.340	0.179	0.140	0.111	0.205	0.087	0.149	

Na, number of alleles; Ne, effective number of alleles; I, Shannon index; Ho, observed heterozygosity; He, expected heterozygosity F, fixation index, an index to evaluate the degree of deviation between the actual observed value and the theoretical value; PIC, polymorphic information content; Prob, P-value; Signif significance (ns indicates not significant, i.e., the population conforms to HWE; * indicates significant difference P<0.05, ** indicates significant difference P<.01, *** indicates significant difference P<0.001).

The observed heterozygosity (Ho) varied from 0.086 to 0.636, while the expected heterozygosity (He) ranged from 0.197 to 0.602. The generally higher values of He compared with Ho suggest a moderate level of heterozygote deficit in some loci, which may be attributed to factors such as population structure, inbreeding, or the presence of null alleles (Weir and Cockerham, 1984). The polymorphism information content (PIC) values ranged from 0.188 to 0.520, indicating that all SSR markers were highly informative and effective for distinguishing genotypes within the studied populations (**Table 1**) (Botstein et al., 1980).

Shannon’s diversity index (I) averaged 0.761 across loci, further supporting the presence of considerable genetic variation within the analyzed germplasm (Nei, 1973). Importantly, no significant linkage disequilibrium (LD) was detected between any pair of loci after Bonferroni correction, indicating that the SSR markers were independently inherited and appropriate for downstream population structure and diversity analyses.

At the population level, genetic diversity indices revealed clear differences among taxa. *P.* purpurea (MX-Z) exhibited moderate genetic diversity (He = 0.330), whereas *P. asperata* populations showed comparatively higher and more variable diversity levels (He = 0.272–0.374) (**Table 2**). Among these, the Mkh-Q population displayed the highest genetic diversity (He = 0.272), suggesting a relatively rich genetic background and potential historical gene flow or large effective population size. In contrast, the MX population showed the lowest diversity (He = 0.374), which may reflect geographic isolation, genetic drift, or localized selection pressures.

**Table 2:** Genetic diversity indices of the population.

Pop		Na	Ne	I	Ho	He	F
BS	Mean	2.667	1.561	0.571	0.332	0.341	0.016
	SE	0.211	0.070	0.047	0.034	0.030	0.050
DTDX	Mean	3.000	1.648	0.596	0.310	0.353	0.103
	SE	0.309	0.115	0.066	0.046	0.042	0.071
JL	Mean	3.267	1.672	0.643	0.386	0.373	-0.035
	SE	0.408	0.105	0.060	0.038	0.034	0.049
MKH	Mean	2.533	1.575	0.508	0.340	0.306	-0.080
	SE	0.256	0.126	0.087	0.069	0.054	0.074
Mkh-Q	Mean	2.533	1.477	0.464	0.289	0.272	-0.017
	SE	0.291	0.112	0.081	0.071	0.049	0.087
MX	Mean	2.800	1.656	0.625	0.358	0.374	0.041
	SE	0.262	0.089	0.047	0.036	0.031	0.056
MX-Z	Mean	3.933	1.897	0.650	0.295	0.330	0.145
	SE	0.511	0.296	0.140	0.066	0.071	0.091
NMX	Mean	3.067	1.628	0.600	0.359	0.352	-0.022
	SE	0.371	0.104	0.064	0.043	0.040	0.045
QL	Mean	3.133	1.605	0.597	0.355	0.350	-0.018
	SE	0.307	0.089	0.052	0.036	0.035	0.038
XBS	Mean	2.867	1.591	0.589	0.352	0.343	-0.028
	SE	0.215	0.090	0.058	0.041	0.037	0.043
XM	Mean	2.867	1.565	0.577	0.350	0.337	-0.044
	SE	0.322	0.082	0.061	0.038	0.035	0.049
XQ	Mean	3.000	1.643	0.609	0.414	0.353	-0.149
	SE	0.276	0.116	0.062	0.052	0.040	0.033
XX	Mean	3.400	1.651	0.634	0.296	0.361	0.145
	SE	0.349	0.101	0.065	0.042	0.040	0.075

Na, observed alleles; Ne, effective alleles; I, Shannon information index; Ho, observed heterozygosity; He, expected heterozygosity; F, fixation.

### 3.2 DNA fingerprinting system validation

All 516 accessions exhibited unique multilocus SSR genotypes across the 15 loci analyzed, confirming the high discriminatory resolution of the selected marker system (**Supplementary Table 3**). This result indicates that the SSR panel is sufficiently robust to distinguish even closely related individuals, thereby providing a reliable framework for germplasm identification and genetic traceability (**Table 1**).

The combined discrimination power (DP) across all loci reached 0.9998, reflecting an extremely high probability of correctly differentiating genotypes within the studied collection. In addition, the probability of identity (PI) for unrelated individuals was estimated at 2.4 × 10^-5^, while the probability of identity among full siblings (PI_sibs) was 4.7 × 10^-3^. These low PI values further demonstrate the strong resolving power of the marker set, particularly in genetically structured or related populations, where discrimination is typically more challenging (Waits et al., 2001; Peakall and Smouse, 2012).

The very low PI values combined with high DP suggest that the probability of two randomly selected individuals sharing identical multilocus genotypes is extremely low. This confirms that the SSR system developed in this study is highly suitable for applications requiring precise individual identification, such as germplasm conservation, breeding program management, and protection of genetic resources. Furthermore, the marker set provides a practical tool for detecting potential sample mislabeling, redundancies, or clonal duplicates within large germplasm collections, thereby improving the efficiency and accuracy of genetic resource curation.

Overall, the validated fingerprinting system demonstrates strong applicability for operational genetic resource management and lays a solid foundation for future molecular breeding and population genetic studies in *Picea* species.

### 3.3 Population structure and species distinction

**Figure 1:**
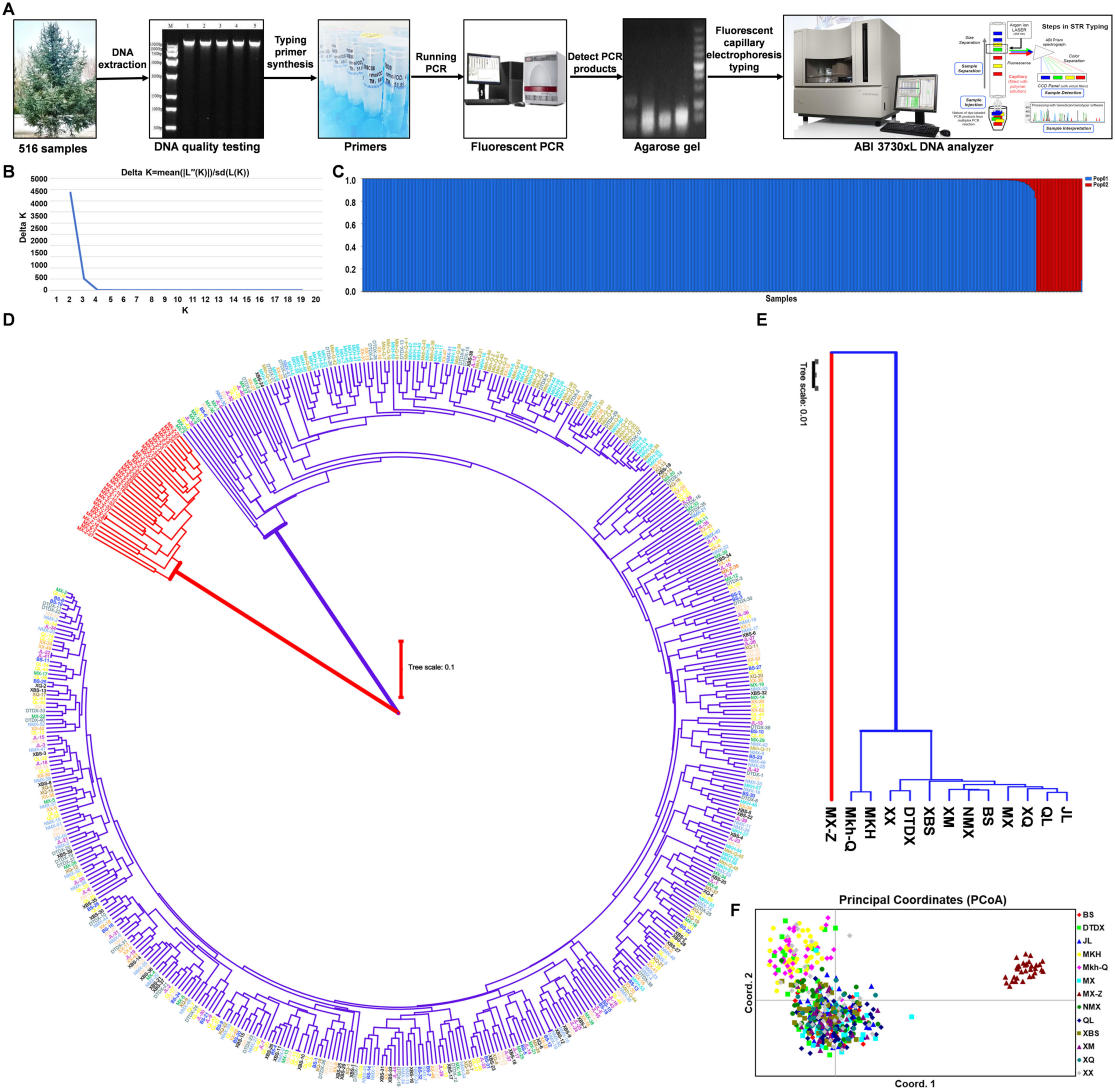
Molecular marker-based identification and population structure analysis of 516 *Picea* accessions from Qinghai Province using 15 fluorescent SSR loci. **(A)** Workflow of SSR genotyping and fingerprinting. The procedure includes DNA extraction, quality assessment, fluorescent PCR amplification with M13-tailed primers, capillary electrophoresis on an ABI 3730xl DNA Analyzer, and allele calling with GeneMapper v5.0. This pipeline generated unique multilocus genotypes for all accessions. **(B)** Estimation of the optimal number of genetic clusters using the ΔK method (Evanno et al., 2005) based on STRUCTURE analysis. The clear peak at K = 2 indicates that the entire dataset is best partitioned into two primary genetic groups, with no support for further sub-structuring. **(C)** STRUCTURE bar plots for K = 2. Each vertical bar represents an individual, and colours denote estimated ancestry proportions from each cluster. At K = 2, the 516 individuals split distinctly into two groups: the blue cluster corresponds exclusively to *Picea purpurea* (population MX-Z), while the red cluster comprises all 12 P*. asperata* populations. **(D)** Neighbour-joining (NJ) phylogenetic tree reconstructed from Nei’s unbiased genetic distance. The tree clearly separates *P. purpurea* (upper clade) from all *P. asperafa* populations (lower clade) with strong bootstrap support (98% at the major split). Scale bar represents genetic distance. **(E)** Principal coordinate analysis (PCoA) based on codominant genetic distances. The first two coordinates explain 28.4% and 19.7% of the total genetic variance, respectively. *P. purpurea* (MX-Z, shown in blue) forms a completely separate cluster along coordinate 1, while all *P. asperafa* populations (shown in red) overlap broadly, confirming the absence of strong geographic or ecological subdivision within this species. **(F)** Principal coordinate analysis (PCoA) based on codominant genetic distances. The first two coordinates explain 28.4% and 19.7% of the total genetic variance, respectively. *P purpurea* (MX-Z shown in blue) forms a completely separate cluster along coordinate 1, while all *P. asperata* populations (shown in red) overlap broadly, confirming the absence of strong geographic or ecological subdivision within this species.

Bayesian model-based clustering analysis revealed that the most likely number of genetic clusters was K = 2, as indicated by the highest ΔK value (**Figures 1A–C**), suggesting a clear hierarchical genetic structure within the studied germplasm. At K = 2, the 516 accessions were distinctly divided into two major genetic groups with minimal admixture. One cluster corresponded exclusively to *Picea purpurea* (population MX-Z), whereas the second cluster (red) included all 12 populations of *Picea asperata*, indicating strong genetic differentiation between the two taxa (**Figure 1C**).

Importantly, no additional biologically meaningful substructure was detected within *P. asperata* across higher K values (K = 3–6) (**Supplementary Figure 2**), suggesting relatively homogeneous genetic background or recent shared ancestry among its populations. This pattern may reflect ongoing or historical gene flow among populations or insufficient time for lineage sorting within the species complex (Pritchard et al., 2000; Evanno et al., 2005).

The results of the Bayesian clustering were strongly supported by complementary multivariate and phylogenetic analyses. The neighbor-joining phylogenetic tree (**Figures 1D, E**) clearly separated *P. purpurea* from all *P. asperata* populations, with high bootstrap support (98%), indicating strong and stable genetic divergence between the two taxa. Similarly, principal coordinates analysis (PCoA) (**Figure 1F**) revealed two clearly separated clusters consistent with the STRUCTURE results, further confirming the presence of two genetically distinct lineages.

The concordance among Bayesian clustering, phylogenetic reconstruction, and PCoA provides robust and convergent molecular evidence supporting the taxonomic distinction between *P. purpurea* and *P. asperata*. These results suggest that the two taxa represent genetically well-differentiated lineages, likely shaped by long-term evolutionary divergence and/or historical geographic isolation. The absence of pronounced internal structure within *P. asperata* further implies a relatively cohesive genetic background across its sampled range, which may be important for future conservation and breeding strategies.

### 3.4 Genetic admixture within *Picea asperata*

Despite the clear species-level genetic separation observed between *Picea purpurea* and *P. asperata*, substantial genetic admixture was detected within *P. asperata* populations. The STRUCTURE analysis (**Figure 1C**) indicated that most individuals of *P. asperata* exhibited largely homogeneous ancestry proportions derived from a single genetic cluster, with only limited individual-level admixture. Importantly, no clear geographic or taxonomically defined subclusters were observed, suggesting weak population structuring across its distribution range (Pritchard et al., 2000; Evanno et al., 2005).

Consistent with this pattern, principal coordinates analysis (PCoA) (**Figure 1F**) revealed strong overlap among individuals from different *P. asperata* populations, further indicating a lack of pronounced spatial genetic differentiation. Similarly, the neighbor-joining phylogenetic tree (**Figure 1E**) failed to resolve well-supported monophyletic groups corresponding to individual populations, suggesting recent divergence and ongoing gene flow among populations within this species complex.

Pairwise genetic differentiation estimates further supported these observations. FST values among *P. asperata* populations were relatively low, ranging from 0.004 to 0.107, indicating weak genetic structure and limited population differentiation. Correspondingly, estimated gene flow (Nm) values were high, ranging from 2.401 to 53.884, suggesting substantial historical and/or contemporary gene exchange among populations (**Supplementary Table 4**). Such levels of gene flow are typically sufficient to counteract the effects of genetic drift and maintain genetic homogeneity across populations (Wright, 1931, 1951).

In contrast, pronounced genetic differentiation was observed between *P. purpurea* and all *P. asperata* populations. Pairwise FST values between the two taxa were high, ranging from 0.324 to 0.453, while corresponding Nm values were consistently below 0.11. These results indicate strong genetic isolation and limited gene exchange between the two species, consistent with long-term reproductive isolation and independent evolutionary trajectories.

### 3.5 Analysis of molecular variance

The analysis of molecular variance (AMOVA) revealed that the majority of genetic variation was distributed within populations, accounting for 17% of the total variation. This high within-population component suggests substantial levels of genetic diversity at the individual level, which is consistent with outcrossing reproductive systems and historically large effective population sizes (Excoffier et al., 1992).

In contrast, 17% of the total genetic variation was attributed to differences among populations within species, indicating moderate but significant population structure within the studied taxa (**Table 3**). This level of differentiation suggests that although gene flow may occur among populations, it is not sufficient to completely homogenize genetic variation across the species range.

**Table 3:** Analysis of molecular variance (AMOVA) of populations.

Source	df	SS	MS	Est. Var.	%
Among Pops	12	577.993	48.166	0.573	17%
Among Indiv	503	1480.153	2.943	0.220	7%
Within Indiv	516	1291.000	2.502	2.502	76%
Total	1031	3349.145		3.295	100%

Source, Source of variation; df, degrees of freedom; SS, total variance; MS, mean square; Est. Var., estimated variance; %, percentage variation; Among Pops, among populations; Among Indiv, among individuals; Within Indiv, within individuals, within-individual variation refers to the genetic variation caused by heterozygous alleles, magnitude of which is related to the number of heterozygous loci in an individual, i.e., the genetic diversity of the individual.

Notably, 10% of the total genetic variation was partitioned between *Picea purpurea* and *P.* asperata, reflecting a statistically significant interspecific component. Although this proportion is lower than the within-population variance, its significance indicates a clear genetic boundary between the two taxa. This result is consistent with the STRUCTURE, PCoA, and phylogenetic analyses, all of which support the presence of two distinct evolutionary lineages. The detected between-species variance further provides robust quantitative evidence for species-level divergence and supports their recognition as genetically differentiated gene pools.

### 3.6 Isolation-by-distance and environmental correlation

Mantel test analysis revealed a weak but statistically significant isolation-by-distance (IBD) pattern across all populations (r = 0.21, p = 0.03), indicating that geographic distance contributes modestly to the observed genetic differentiation. However, the relatively low correlation coefficient suggests that spatial separation explains only a small proportion of the genetic variation (approximately 4%), implying that dispersal and gene flow may occur over relatively long distances in this species complex. Such weak IBD patterns are commonly observed in long-lived wind-pollinated tree species, where pollen and seed dispersal can partially counteract the effects of geographic distance on genetic differentiation (Mantel, 1967; Wright, 1943).

To further disentangle the potential drivers of genetic structure, partial Mantel tests were conducted to assess the relationship between genetic and environmental distances while controlling for geographic distance. The results showed no significant correlation between genetic distance and environmental distance (r = 0.09, p = 0.24), indicating that the genetic structure is not strongly associated with the examined environmental variables, including elevation, temperature, and precipitation. This suggests that isolation-by-environment (IBE) is unlikely to be a major factor shaping the observed genetic patterns in the studied populations.

The lack of a strong environmental signal, combined with the weak IBD pattern, suggests that neutral evolutionary processes such as historical gene flow, genetic drift, and shared ancestry are likely the primary drivers of the current genetic structure. In particular, the relatively weak spatial genetic structure is consistent with the high gene flow inferred from FST and Nm estimates, further supporting the idea of extensive historical connectivity among populations within *Picea asperata*.

### 3.7 Elevation−linked genetic diversity patterns

To evaluate the relationship between genetic diversity and elevation, we correlated population−level expected heterozygosity (He) with the mean elevation of each sampling site. A significant negative correlation was detected (**Supplementary Figure 1**; **Supplementary Table 1**) (Pearson’s r = -0.67, *p* = 0.012), indicating that higher−altitude populations tend to harbor lower genetic diversity.

When populations were grouped into three elevation classes: low (<2800 m, n = 4 populations), mid (2800–3200 m, n = 5 populations), and high (>3200 m, n = 4 populations), clear differences emerged. Mean He values were 0.363 ± 0.015, 0.342 ± 0.018, and 0.309 ± 0.021 for low−, mid−, and high−elevation groups, respectively, with significant differences between low and high groups (Tukey’s HSD, *p* = 0.018). Similarly, allelic richness (Na) declined from 3.12 ± 0.18 in low−elevation populations to 2.73 ± 0.21 in high−elevation populations. Shannon’s diversity index (I) showed a parallel decreasing trend (0.623 to 0.567).

These results demonstrate that the 15 SSR markers effectively captured elevational gradients in genetic diversity, with the largest differences observed between low− and high−elevation populations. The consistent decline across multiple diversity indices suggests that elevation is a meaningful factor shaping genetic variation in *Picea* populations in Qinghai Province, possibly reflecting reduced effective population sizes, historical bottlenecks, or stronger genetic drift in high−altitude environments.

## 4 Discussion

### 4.1 Resolving taxonomic ambiguity: *P. purpurea* as a distinct lineage

A key contribution of this study is the robust molecular resolution of the long-standing taxonomic ambiguity between *Picea purpurea* and *Picea asperata*. By integrating a large sample size with multiple complementary analytical approaches (STRUCTURE, phylogenetic reconstruction, PCoA, and FST-based differentiation), we consistently demonstrate that *P. purpurea* represents a genetically distinct evolutionary lineage.

The high genetic differentiation observed between the two taxa, combined with low estimated gene flow and a significant between-species component in AMOVA, provides strong and convergent evidence for species-level divergence (Excoffier et al., 1992; Wright, 1951). These results are further consistent with previous phylogenomic studies (Shen et al., 2019b; Ran et al., 2018b), while providing finer-scale resolution using population-level SSR markers.

Importantly, despite partial morphological overlap reported in earlier taxonomic treatments, *P. purpurea* retains a clear and stable genetic identity. This finding has important conservation implications, as *P. purpurea* should be treated as an Evolutionarily Significant Unit (ESU) to preserve its unique genetic resources and evolutionary potential (Moritz, 1994).

### 4.2 High gene flow and admixture within *Picea asperata* populations

In contrast to the strong interspecific divergence, *P. asperata* populations in Qinghai Province exhibit extensive genetic admixture and weak population structure. This is supported by low pairwise FST values (0.004–0.107), high gene flow estimates (Nm=2.401–53.884), overlapping PCoA distributions, and the absence of well-supported population-specific clades in phylogenetic analyses.

Together, these results indicate a genetically interconnected metapopulation system. Several non-exclusive processes may explain this pattern, including wind-mediated pollen dispersal typical of conifers, relatively continuous habitat distribution facilitating gene exchange, and historical connectivity following post-glacial recolonization events (Petit et al., 2003; Hamrick, 2004). This pattern is consistent with findings from other parts of the world, where studies have shown that recurrent hybridization and gene flow, shaped by multiple glacial cycles, have had a profound impact on the evolutionary history of spruce species (Zhou et al., 2023).

Although Mantel tests indicated a weak but significant isolation-by-distance pattern, its explanatory power is limited, suggesting that spatial distance only partially constrains gene flow. Notably, our estimates of population differentiation are lower than those reported in earlier studies of *P. asperata* (Wang et al., 2006), which likely reflects differences in sampling scale and geographic coverage. This highlights the importance of regional-scale genetic assessments for accurately characterizing population connectivity and informing conservation planning.

### 4.3 A validated DNA fingerprinting system for operational use

We developed and validated a 15-locus SSR fingerprinting system with extremely high discriminatory power and very low probability of identity, demonstrating strong applicability for individual-level identification and traceability.

To our knowledge, this represents the first validated molecular fingerprinting system specifically designed for Qinghai spruce germplasm resources. The high resolution of the marker set makes it suitable for multiple applied purposes, including germplasm authentication, breeding management, and conservation monitoring.

In operational contexts, this system enables: (i) accurate verification of planting materials to prevent mislabeling and genetic contamination; (ii) lineage tracking in breeding and restoration programs; and (iii) support for provenance certification and genetic resource protection. Although long-term application will require continuous database updating and quality control, the current validation provides a robust theoretical and practical foundation for implementation in forest genetic resource management (Waits et al., 2001).

### 4.4 Comparison with global *Picea* studies

Our findings are broadly consistent with global patterns of genetic structure observed in *Picea* species. High gene flow and low population differentiation are commonly reported in widely distributed spruce species such as *P. abies* and *P. glauca*, where extensive pollen dispersal and historical connectivity maintain genetic cohesion (Hamilton et al., 2013; Tollefsrud et al., 2008).

However, the very low differentiation observed among *P. asperata* populations (FST values 0.004–0.107) is at the lower end of values reported for conifers, indicating exceptionally high connectivity in the Qinghai region. In contrast, the interspecific divergence between *P. purpurea* and *P. asperata* (FST values 0.324 to 0.453) is comparable to divergence levels observed between other sympatric or closely related spruce species pairs (De La Torre et al., 2015).

The elevation-associated diversity gradient further aligns with findings in Asian spruces such as *P. crassifolia* and *P. koraiensis*, suggesting that topographic gradients may represent a general driver of intraspecific genetic variation in montane conifer systems (Feng et al., 2023; Sun et al., 2023). Similarly, whole-genome level investigations on other Chinese spruces, such as *P. meyeri* and *P. mongolica*, have revealed significant adaptive divergence and historical population dynamics, underscoring the complex evolutionary history of the genus in China (Liu et al., 2024).

### 4.5 Conservation and management recommendations

Based on our findings, we propose the following conservation strategies:

For *Picea purpurea*, its status as a distinct ESU warrants priority conservation. *Ex situ* conservation efforts, including germplasm banking and seed orchard establishment, should be implemented. Importantly, mixing with *P. asperata* should be strictly avoided in restoration programs to prevent genetic swamping and erosion of unique genetic diversity (Moritz, 1994).

For *Picea asperata*, maintaining landscape connectivity is essential to preserve high levels of gene flow and genetic cohesion. Seed transfer zones should be designed to reflect natural connectivity patterns, while conserving populations across elevational gradients to capture maximum genetic diversity.

The validated SSR fingerprinting system provides a practical tool for routine monitoring, enabling certification of seed sources and supporting sustainable forest management practices.

### 4.6 Limitations and future directions

Several limitations should be acknowledged. First, SSR markers, while effective for population structure and fingerprinting, are neutral and cannot directly detect adaptive genetic variation. Second, gene flow estimates (Nm) assume equilibrium conditions, which may not fully reflect post-glacial demographic dynamics. Third, the observed elevation–diversity relationship is correlative and does not establish causality.

Future research should employ genome-wide SNP datasets (GBS or whole-genome resequencing) to identify adaptive loci associated with environmental gradients (Di Pierro et al., 2017). In addition, common garden and reciprocal transplant experiments are needed to validate potential adaptive differentiation. Expanding sampling across broader geographic regions will also improve understanding of regional connectivity and historical demographic processes.

### 4.7 Efficiency of SSR markers in detecting elevation−linked diversity

An important finding of this study is the significant negative correlation between genetic diversity (He) and elevation, which aligns with patterns observed in other montane conifer systems, including *Picea crassifolia* and *Picea koraiensis* (Feng et al., 2023; Sun et al., 2023). Despite being neutral markers, the 15 SSR loci proved efficient in detecting this elevational cline, likely due to three factors: (1) their high polymorphism (mean PIC = 0.363) provided sufficient resolution to capture subtle differences among populations; (2) the large sample size (516 accessions) and broad elevational range (2120–3600 m) offered strong statistical power; and (3) the multilocus nature of SSR data enables robust estimation of diversity parameters even in the absence of adaptive markers.

The observed pattern, lower diversity at higher elevations, may reflect several non−exclusive processes. High−elevation populations often have smaller effective population sizes (Ne) due to limited habitat availability, increasing the impact of genetic drift (Jump and Peñuelas, 2005). Additionally, historical bottlenecks during Pleistocene glacial cycles may have disproportionately affected high−altitude refugial populations, with subsequent recolonization further reducing diversity (Petit et al., 2003; Körner, 2007). While SSR markers cannot distinguish between these scenarios, they provide a valuable baseline for identifying populations of conservation concern.

Importantly, the efficiency of our SSR panel in detecting this elevation−diversity association demonstrates that even neutral markers, when applied at sufficient scale, can reveal biologically meaningful environmental patterns. This finding has practical implications: SSR−based screening could serve as a cost−effective first−step approach for identifying priority populations for conservation and genomic characterization in other montane tree species.

## 5 Conclusion

This study developed and validated a robust 15−SSR fingerprinting system for 516 *Picea* accessions from Qinghai Province. The marker panel exhibited high discriminatory power and extremely low probability of identity, making it a reliable tool for germplasm identification, lineage tracing, and seed source certification. Integrated population genetic analyses clearly resolved the long−standing taxonomic ambiguity between *Picea purpurea* and *Picea asperata*, demonstrating that *P. purpurea* represents a genetically distinct lineage. In contrast, *P. asperata* populations showed extensive admixture and high gene flow, indicating a genetically cohesive metapopulation. Notably, we detected a significant negative correlation between genetic diversity (He) and elevation, revealing an elevation−linked diversity cline that has direct implications for conservation prioritization under climate change. Overall, our findings provide strong molecular evidence for species delimitation in *Picea*, establish a practical genetic toolkit for sustainable forest management, and contribute to understanding environment−associated genetic variation in montane conifer systems.

## Data Availability

The original contributions presented in the study are included in the article/**Supplementary Material**, further inquiries can be directed to the corresponding author/s.

## Supplementary material

Supplementary Figure 1 13 *Picea* populations was collected from Qinghai Province, China. The small red flag represents the sampling location.

Supplementary Figure 2 Structure bar plots for K = 3-6. Each vertical bar represents an individual, and colors denote inferred ancestry proportions.
