# Supplementary figures and images for: Large−scale SSR fingerprinting resolves taxonomic distinction between Picea purpurea and Picea asperata and reveals elevation−linked diversity in Qinghai Province, China
Source: Front Plant Sci. 2026 Jul 10;17:1882848. doi: 10.3389/fpls.2026.1882848 (PMC13396211; doi:10.3389/fpls.2026.1882848)

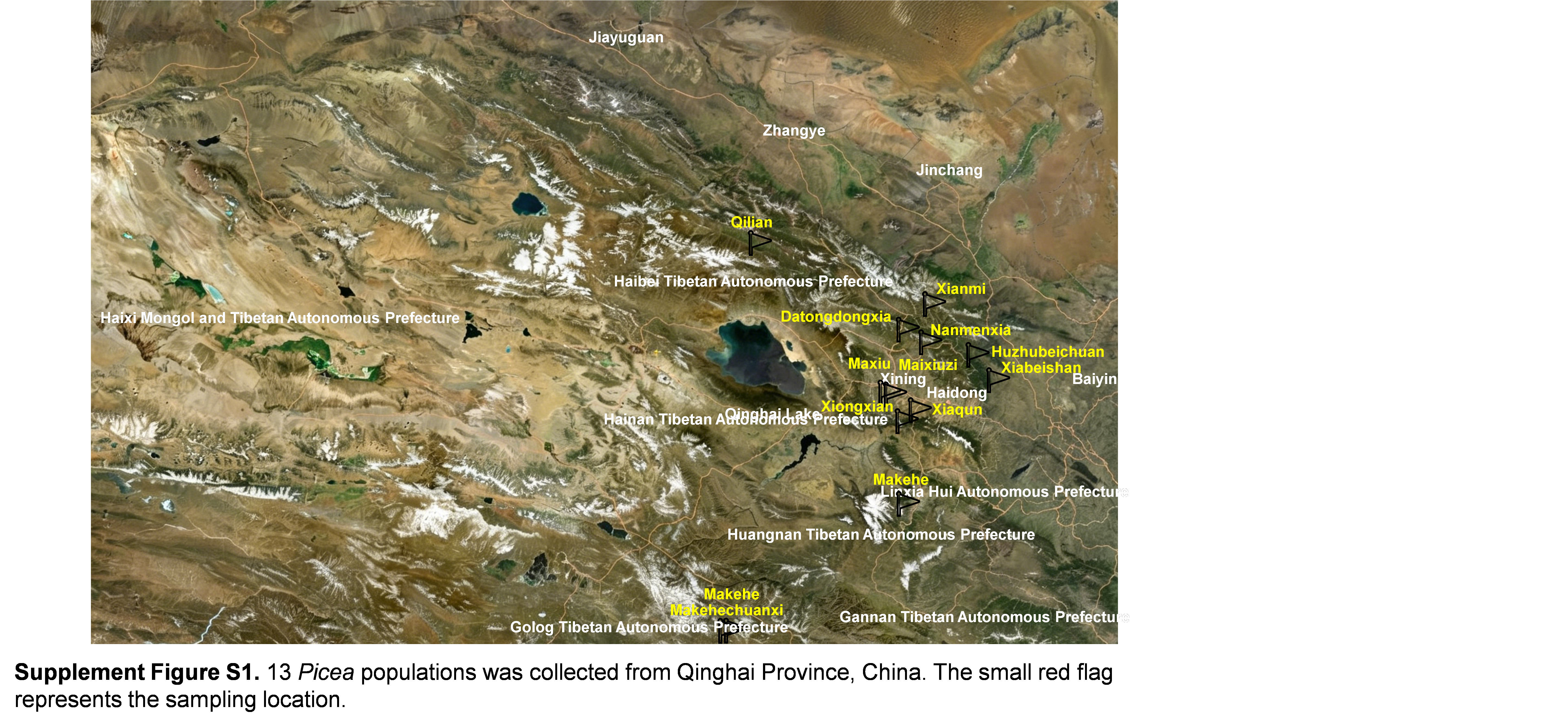

Supplement: Supplementary Figure 1 — 13 Picea populations was collected from Qinghai Province, China. The small red flag represents the sampling location. [file Image1.tif]

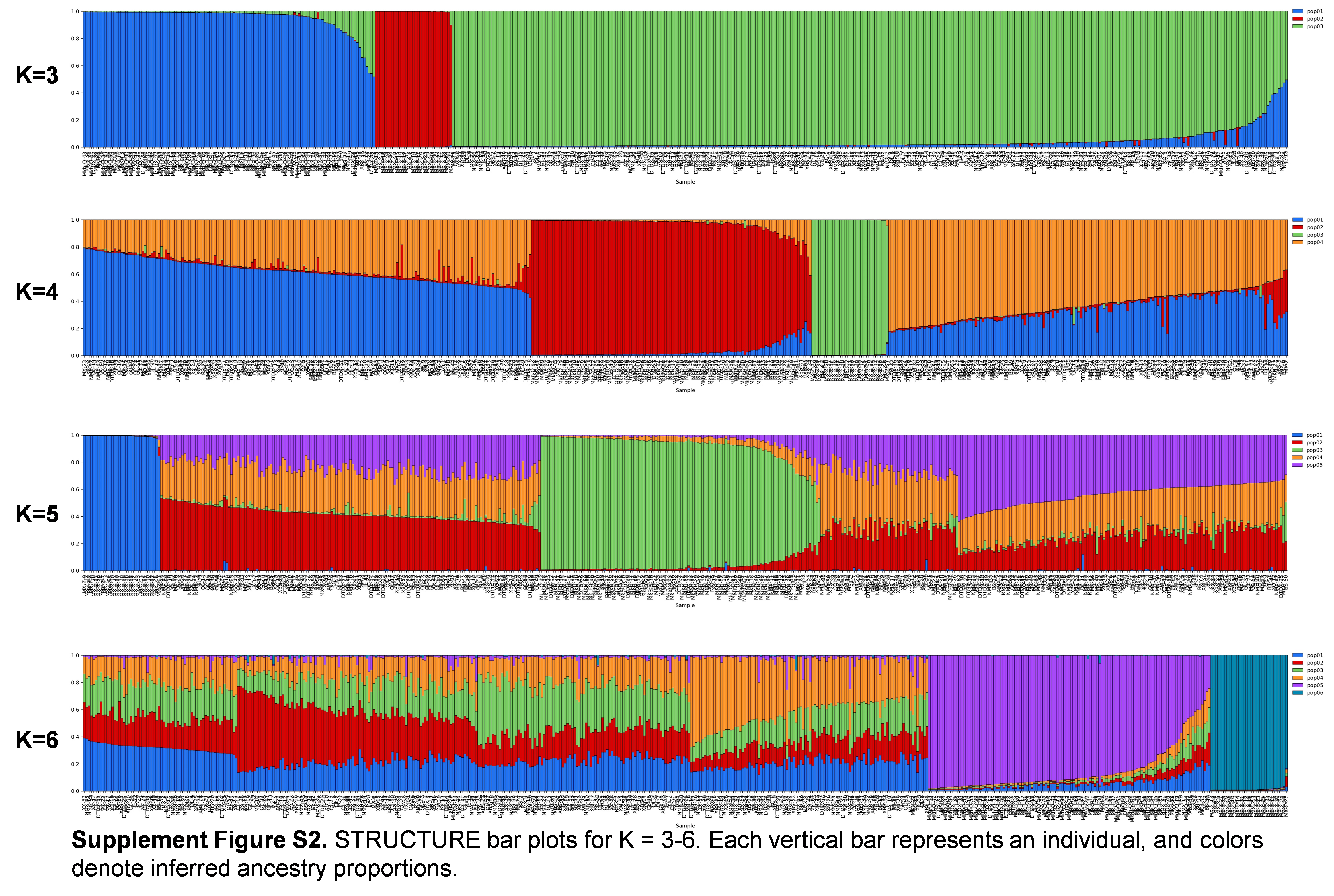

Supplement: Supplementary Figure 2 — Structure bar plots for K = 3-6. Each vertical bar represents an individual, and colors denote inferred ancestry proportions. [file Image2.tif]
